# Supplementary material for: HIV-1 promoter is gradually silenced when integrated into BACH2 in Jurkat T-cells
Source: PeerJ. 2020 Nov 24;8:e10321. doi: 10.7717/peerj.10321 (PMC7694569; doi:10.7717/peerj.10321)
Supplement: Supplemental Information 1 [file peerj-08-10321-s001.docx]

##### **Supplementary Material**

**Supplementary Table 1:** Primers used to amplify the BACH2 and AAVS1 homologous arms, to amplify and sequence the junction between the vector LTatCL[M] and BACH2 or AAVS1, to test for mono- or bi-allelic integration, and to amplify the vector LTatCL[M].

| **Primer name** | **Primer sequence (5‘-3‘)** | **Description** |
| --- | --- | --- |
| BsrGI_BACH2_1_5fw | NNNN*TGTACA*CACTTTCGTGGAGCTGTTGTTAG | BsrGI overhang, forward primer, cloning of *BACH2_*i5 5` homologous arm |
| PacI_BACH2_1_5rc | NNNN*TTAATTAA*TTCTGGGTTCTGTTACTCAC | PacI overhang, reverse primer, cloning of *BACH2_*i5 5` homologous arm |
| AscI_BACH2_1_3fw | NNNN*GCGCGCC*TAGCATGGAGGAGTATGACAG | AscI overhang, forward , cloning of *BACH2_*i5 3` homologous arm |
| SpeI_BACH2_1_3rc | NNNNNN*ACTAGT*GCATTAGAAAGTGTTTCCAATCC | SpeI overhang, reverse primer, cloning of *BACH2_*i5 3` homologous arm |
| BsrGI_BACH2_2_5fw | NNNN*TGTACA*GTAACAGTGCTCTCTATACATTATCC | BsrGI overhang, forward primer, cloning of *BACH2_*i2 5` homologous arm |
| PacI_BACH2_2_5rc | NNNNNN*TTAATTAA*CTATGGTTTCACCTTTTCTGGATATTTC | PacI overhang, reverse primer, cloning of *BACH2_*i2 5` homologous arm |
| AscI_BACH2_2_3fw | NNNN*GGCGCGCC*ACGCAGGAAGCAGAGAAGTG | AscI overhang, forward primer, cloning of *BACH2_*i2 3` homologous arm |
| SpeI_BACH2_2_3rc | NNNNNN*ACTAGT*CTACTTTGATATTTGAAGGATAGATTCC | SpeI overhang, reverse primer, cloning of *BACH2_*i2 3` homologous arm |
| BsrG1_AAVS1_5arm_fw | NNNN*TGTACA*GGTCCTGCTTTCTCTGACCTGC | BsrGI overhang, forward primer, cloning of AAVS1 5` homologous arm |
| Pac1_AAVS1_5arm_rc | NNNN*TTAATTAA*TGTCCCTAGTGGCCCCACTGTG | PacI overhang, reverse primer, cloning of AAVS1 5` homologous arm |
| Asc1_AAVS1_3arm_fw | NNNN*GGCGCGCC*GGATTGGTGACAGAAAAGCCCCATC | AscI overhang, forward primer, cloning of AAVS1 3` homologous arm |
| Spe1_AAVS1_3arm_rc | NNNNNN*ACTAGT*GTCTGAAGAGCAGAGCCAGGAACC | SpeI overhang, reverse primer, cloning of AAVS1 3` homologous arm |
| geneB21_fw | ATACAAGGAAACCACAGCCTTCTGG | outer/inner PCR of junction, mono- or bi-allelic integration, and vector amplification and sequencing of LTatCL[M]/*BACH2_*i5s and LTatCL[M]/*BACH2_*i5c |
| REV_Tat_rc | TCTAGTCTAGGATCTACTGGCTCC | outer PCR of junction LTatCL[M]/*BACH2*_i5c, and inner PCR of junction LTatCL[M]/*BACH2*_i2c |
|  |  |  |
| nB21_fw | CAGCAACATAAGCATCCCAAGTTGATG | inner PCR of junction LTatCL[M]/*BACH2*_i5c, and inner PCR of junction LTatCL[M]/*BACH2*_i5s |
|  |  |  |
| 5’LTRIII | TGTGGTAGATCCACAGATCAAG | outer PCR of junction LTatCL[M]/AAVS1_c, and inner PCR of junction LTatCL[M]/*BACH2*_i5c and LTatCL[M]/AAVS1_c |
|  |  |  |
| KYL-INS3LTR-1897fw | GTCAACATCAAGTTGGACATCACC | outer PCR of junction LTatCL[M]/AAVS1_s, and inner PCR of junction LTatCL[M]/*BACH2*_i5s |
|  |  |  |
| PolyA_RO_rc | TGTGTCTAGAGCTCGAGCATGC | inner PCR of junction, and vector amplification and sequencing of LTatCL[M]/*BACH2_*i5s, LTatCL[M]/*BACH2_*i2s and LTatCL[M]/AAVS1*_*s |
| geneB22_fw | ATACAAGGAAACCACAGCCTTCTGG | outer PCR of junction, mono- or bi-allelic integration, vector amplification and sequencing of LTatCL[M]/*BACH2_*i2s and LTatCL[M]/*BACH2_*i2c |
| SigmaP1_fw | GGCCCTGGCCATTGTCACTT | outer PCR of junction LTatCL[M]/AAVS1*_*s and LTatCL[M]/AAVS1*_*c |
| LJ_AAVS1_fw3 | CTTTGAGCTCTACTGGCTTCTGC | inner PCR of junction and mono- or bi-allelic integration LTatCL[M]/AAVS1*_*s and LTatCL[M]/AAVS1*_*c |
| A.I_BACH2.1_3'arm_rc | AGACTGGCTGTCATACTCCTCCATG | mono- or bi-allelic integration of LTatCL[M]/*BACH2*_i5s and LTatCL[M]/*BACH2*_i5c |
|  |  |  |
| AI_AAVS1_3'arm1Rv | GCCTAAGGATGGGGCTTTTCTG | mono- or bi-allelic integration of LTatCL[M]/AAVS1_s and LTatCL[M]/AAVS1_c |
|  |  |  |
| A.I_BACH2.2_3'arm_rc | GAAACCACTTCTCTGCTTCCTGC | mono- or bi-allelic integration of LTatCL[M]/*BACH2*_i2s and LTatCL[M]/*BACH2*_i2c |
|  |  |  |
|  |  |  |
| KYL_5LTRFL_1121rc | GGCACGCGTCTAATCGAATGG | vector amplification and sequencing of LTatCL[M]/*BACH2_*i5s, LTatCL[M]/*BACH2_*i5c, LTatCL[M]/*BACH2*_i2s and LTatCL[M]/*BACH2_*i2c |
| PolyA_fw | GCATGCTCGAGCTCTAGACACA | vector amplification and sequencing of LTatCL[M]/*BACH2_*i5s, LTatCL[M]/*BACH2_*i5c, LTatCL[M]/*BACH2_*i2s and LTatCL[M]/*BACH2_*i2c |
| Rev_Tat_fw | GGAGCCAGTAGATCCTAGACTAGA | vector amplification and sequencing of LTatCL[M]/*BACH2_*i5s, LTatCL[M]/*BACH2_*i5c, LTatCL[M]/*BACH2_*i2s and LTatCL[M]/*BACH2_*i2c |
| gBACH2_1_Fw | CACCGATACTCCTCCATGCTATTCT | *BACH2_*i5 gRNA (cloning of guide sequence into pX458) |
| gBACH2_1_rc | AAACAGAATAGCATGGAGGAGTATC | gRNA of *BACH2_*i5 (cloning of guide sequence into pX458) |
| gBACH2_2_fw | CACCGAAAGGTGAAACCATAGACGC | gRNA of *BACH2_*i2 (cloning of guide sequence into pX458) |
| gBACH2_2_rc | AAACGCGTCTATGGTTTCACCTTTC | gRNA of *BACH2_*i2 (cloning of guide sequence into pX458) |
| gAAVS1_fw | CACCGGGGCCACTAGGGACAGGAT | gRNA of AAVS1 (cloning of guide sequence into pX458) |
| gAAVS1_rc | AAACATCCTGTCCCTAGTGGCCCC | gRNA of AAVS1 (cloning of guide sequence into pX458) |
| BaEx7-Fw | GAACCAACTCCAGTGACGAATCC | mRNA quantification downstream of *BACH2*_i2 and *BACH2*_i5 |
| BaEx8-Rv | CTAACTGTTCTGAGGTTAGCTTGTGC | mRNA quantification downstream of *BACH2*_i2 and *BACH2*_i5 |
| Mf45 | TCGACAGTSAGCCGCATCTT | mRNA quantification of Glycerinaldehyd-3-phosphat-Dehydrogenase (GAPDH) |
| Mf46 | GGCAACAATATCCAGTTTACCAG | mRNA quantification of Glycerinaldehyd-3-phosphat-Dehydrogenase (GAPDH) |


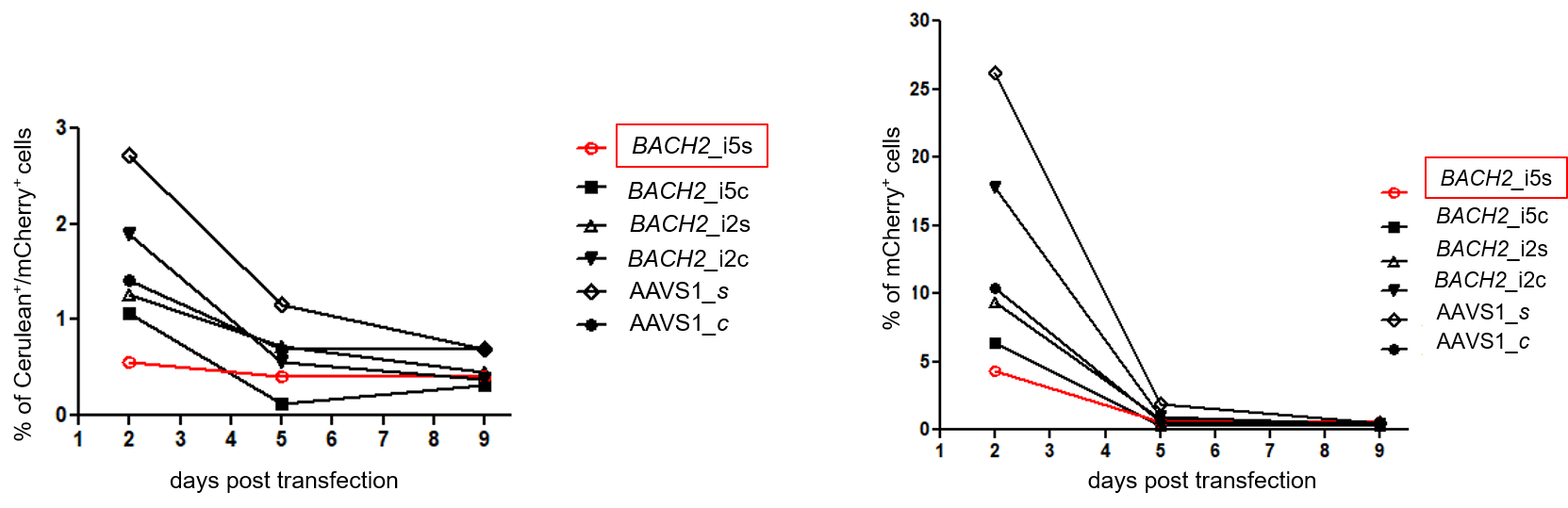


**Supplementary Figure 1: FACS analysis of Cerulean^+^/mCherry^+^ and single mCherry^+^ expression 2, 5, and 9 days post transfection.** Percentage of Cerulean^+^/mCherry^+^ (left panel) and single mCherry^+^ (right panel) expressing cells over time in all six cell variants each, transfected with one of the six vectors LTatCL[M], are shown for one exemplary experiment. Each symbol represents one cell variant, open symbols represent cells in which LTatCL[M] is integrated in the same transcriptional orientation of the gene, closed symbols represent cells in which LTatCL[M] is integrated in the convergent transcriptional orientation of the gene. The experiment was carried out three times independently. The *in vivo* observed preferential HIV-1 integration loci in *BACH2, BACH2*_i5s, is highlighted by red boxes.


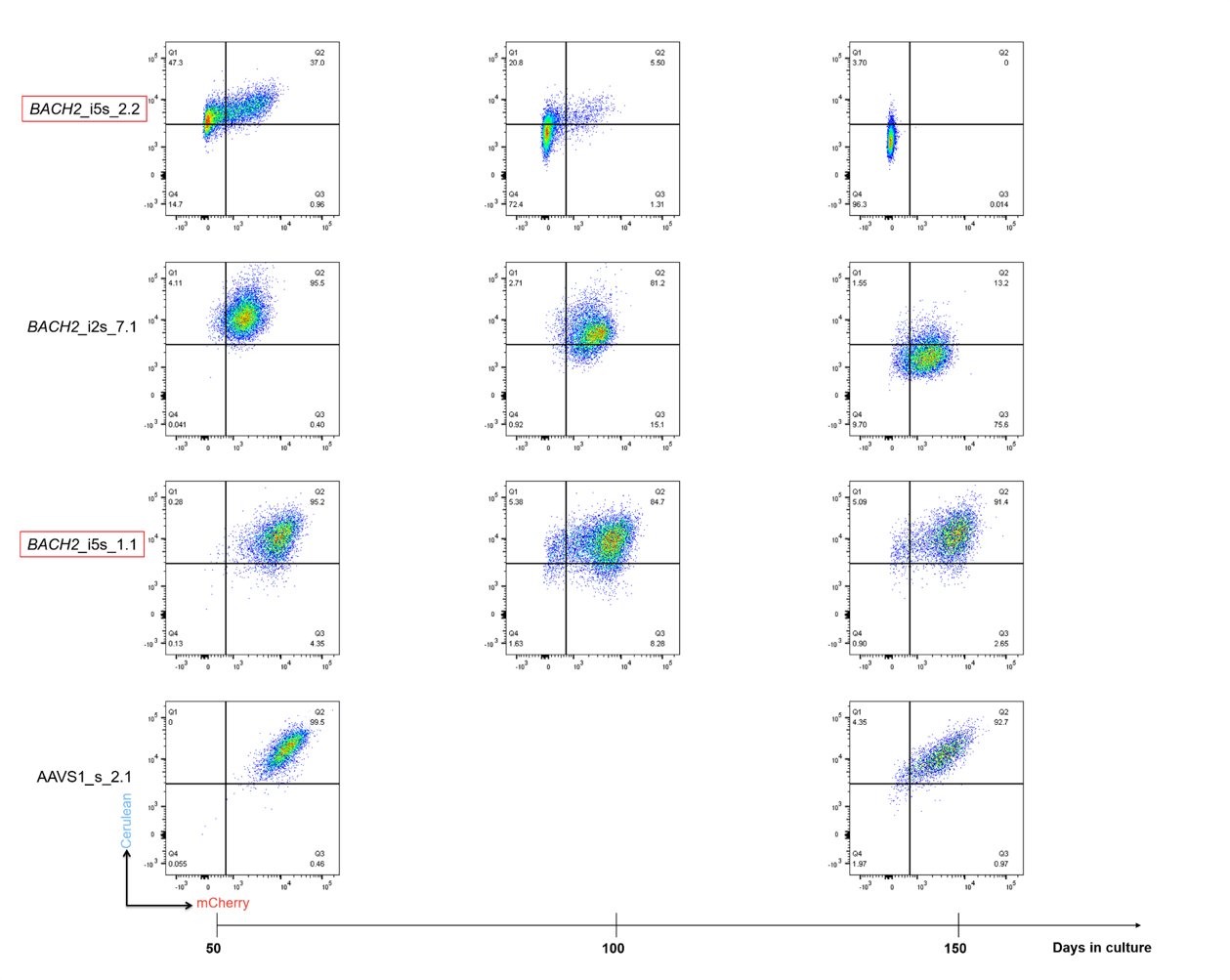


**Supplementary Figure 2: Longitudinal FACS analysis of Cerulean^+^/mCherry^+^ monoclonal cell lines for up to 162 days.** Three time points (50, 100, and 150 days in culture) are depicted for exemplary Cerulean^+^/mCherry^+^ monoclonal cell lines showing different phenotypic changes over time; Cerulean^+^/mCherry^+^ to a Cerulean^-^/mCherry^-^ expressing phenotype depicted for *BACH2*_i5s_2.2, Cerulean^+^/mCherry^+^ to single mCherry^+^ expressing phenotpye depicted for *BACH2*_i2s_7.1 and Cerulean^+^/mCherry^+^ monoclonal cell lines maintaining Cerulean^+^/mCherry^+^ expressing phenotype depicted for *BACH2*_i5s_1.1 and AAVS1_s_2.1. The experiment was carried out two times independently. The *in vivo* observed preferential HIV-1 integration loci in *BACH2, BACH2*_i5s, is highlighted by red boxes.


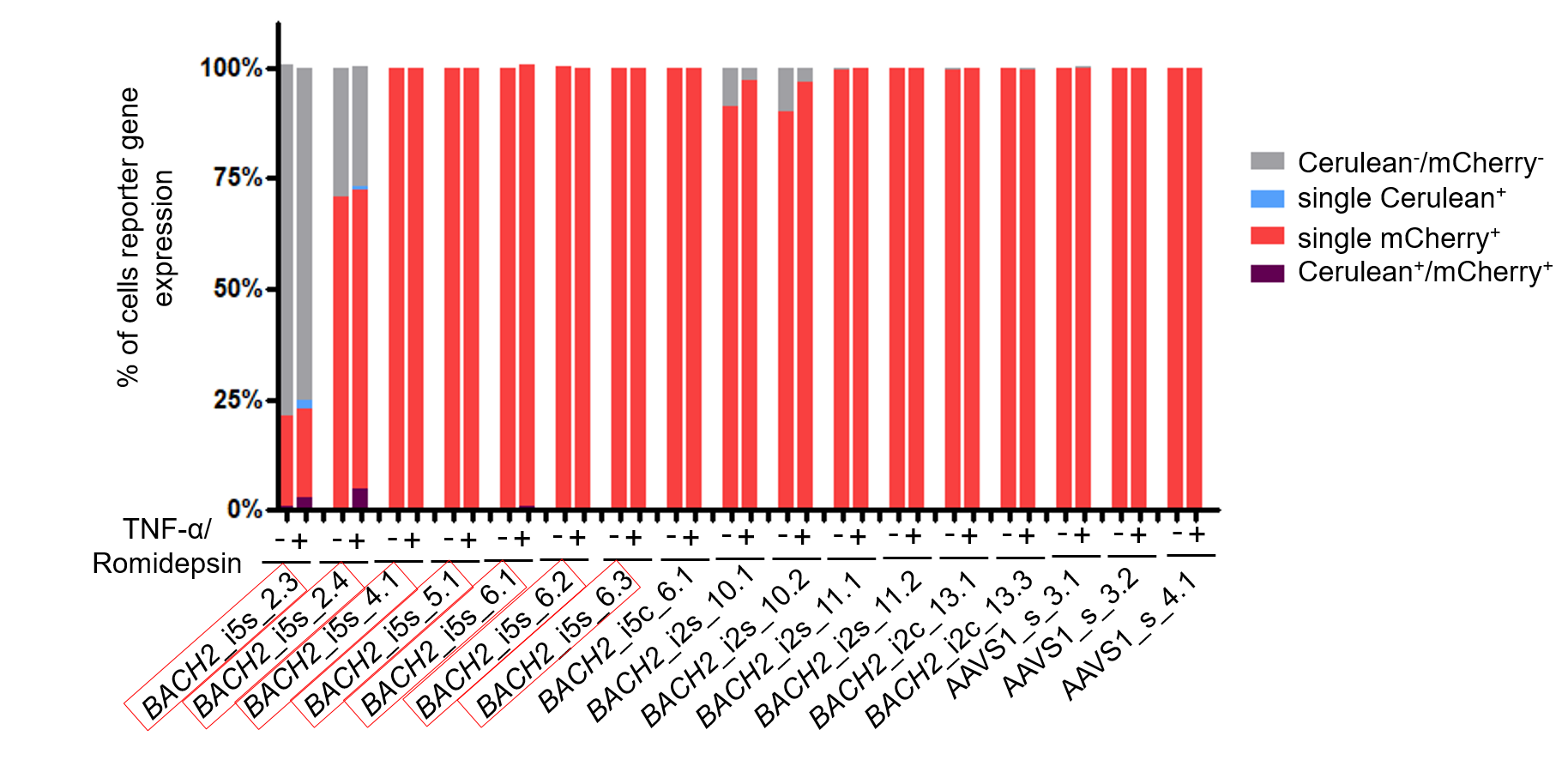


**Supplementary Figure 3:** **Treatment of single mCherry^+^ cell clones with TNF-α and Romidepsin.** Monoclonal cell lines were treated with 10 ng/μL TNF-α and 4 nM Romidepsin for 24 hours followed by FACS analysis. Two independent experiments were performed. The *in vivo* observed HIV-1 integration loci in *BACH2*, *BACH2*_i5s, is highlighted by red boxes.


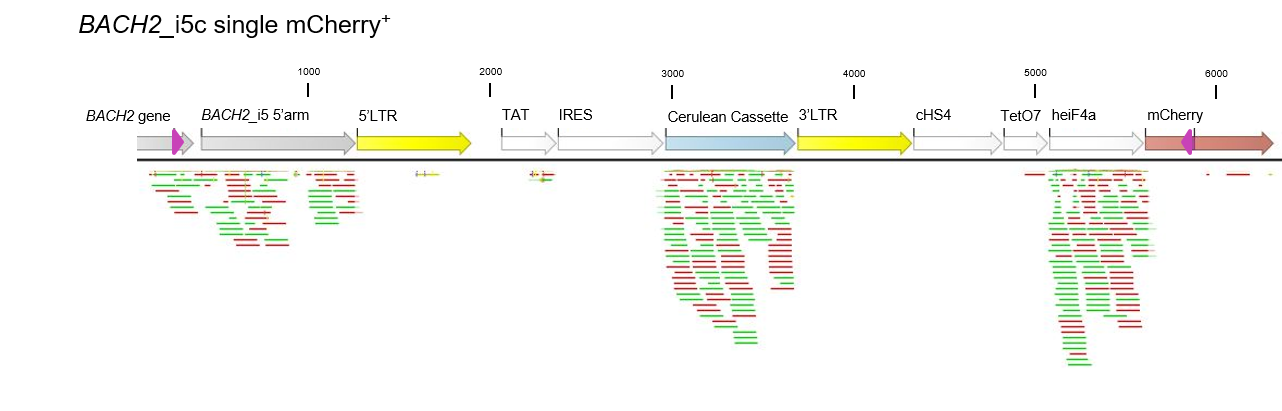


**Supplementary Figure 4:** **Mapping of large internal deletions within LTatCL[M] in single mCherry^+^ cell population after 1^st^ bulk sort.** Deletions in the integrated vector LTatCL[M] are depicted for cell population of bulk sorted *BACH2*_i5c of single mCherry^+^ cells. Pink arrow indicates primer chosen to amplify the vector.


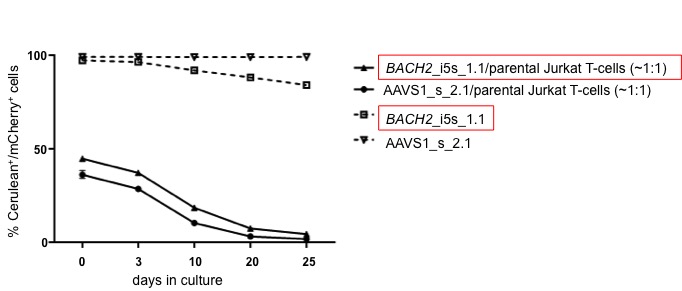


**Supplementary Figure 5:** **Outgrowth of parental Jurkat T-cell line within 25 days in a cell-growth competition experiment with Cerulean^+^/mCherry^+^ monoclonal cell lines.** Five time points (0, 3, 10, 20, 25 days in culture) are depicted for exemplary Cerulean^+^/mCherry^+^ monoclonal cell lines, *BACH2*_i5s_1.1 and AAVS1_s_2.1, mixed in an approximately 1:1 ratio with the parental Jurkat T-cell line and *BACH2*_i5s_1.1 and AAVS1_s_2.1 without addition of the parental Jurkat T-cell line. Each data point represents the mean of three independent cell-growth competition assays (n=3) and error bars depict standard error means. Some error bars are within data points. The *in vivo* observed preferential HIV-1 integration loci in *BACH2, BACH2*_i5s, is highlighted by red boxes.
